# Cold exposure and thermoneutrality similarly reduce supraclavicular brown adipose tissue fat fraction in fasted young lean adults

**DOI:** 10.1096/fj.202402415R

**Published:** 2025-01-11

**Authors:** Robin van Eenige, Carlijn A. Hoekx, Aashley S. D. Sardjoe Mishre, Maaike E. Straat, Mariëtte R. Boon, Borja Martinez‐Tellez, Patrick C. N. Rensen, Hermien E. Kan

**Affiliations:** ^1^ Division of Endocrinology, Department of Medicine Leiden University Medical Center Leiden The Netherlands; ^2^ Einthoven Laboratory for Experimental Vascular Medicine Leiden University Medical Center Leiden The Netherlands; ^3^ Department of Radiology, C.J. Gorter MRI Center Leiden University Medical Center Leiden The Netherlands; ^4^ Department of Nursing Physiotherapy and Medicine, SPORT Research Group (CTS‐1024), CIBIS Research Center University of Almería Almería Spain; ^5^ Biomedical Research Unit Torrecárdenas University Hospital Almería Spain; ^6^ CIBER de Fisiopatología de la Obesidad y Nutrición (CIBEROBN) Instituto de Salud Carlos III Granada Spain

**Keywords:** brown adipose tissue, cold exposure, fat fraction, magnetic resonance imaging

## Abstract

Brown adipose tissue (BAT) is a metabolically highly active tissue that dissipates energy stored within its intracellular triglyceride droplets as heat. Others have previously utilized MRI to show that the fat fraction of human supraclavicular BAT (scBAT) decreases upon cold exposure, compared with baseline (i.e., pre‐cooling). However, comparisons to a control group that was not exposed to cold are largely lacking. We recently developed a non‐invasive dynamic MRI protocol that allows for quantifying scBAT fat fraction changes over time. Here, we aimed to study the effect of cold exposure versus thermoneutrality on fat fraction changes in human scBAT. Ten young (mean age: 21.5 ± 0.7 years), lean (mean BMI: 21.7 ± 0.5 kg/m^2^), 12 h‐fasted volunteers (9 females; 1 male) underwent up to 70 consecutive MRI scans each on two separate study visits in a cross‐over design. Participants were exposed to a temperature of 32°C for 10 scans (i.e., ±16 min), which was then either lowered to 18°C (i.e., cold exposure) or was maintained at 32°C (i.e., thermoneutrality). Dynamic fat fraction changes were quantified, and self‐reported thermal perception scores were monitored. The fat fraction in scBAT decreased over time upon cold exposure (*r* = −.222, *p* < .001). Interestingly however, we also observed a decrease in scBAT fat fraction over time upon thermoneutrality (*r* = −.212, *p* < .001). No difference was observed between the two temperature conditions (*p* = .55), while self‐reported thermal perception scores were consistently higher (i.e., colder) upon cold exposure. In the trapezius muscle and the humerus bone as control tissues, the fat fraction was unaffected in both temperature conditions. The fat fraction in subcutaneous white adipose tissue (sWAT) however, also decreased over time upon cold exposure (*r* = −.270, *p* < .001) and during thermoneutrality (*r* = −.190, *p* < .001), again with no difference (*p* = .92) between the two temperature conditions. In conclusion, our results show that in 12 h‐fasted, healthy individuals cold exposure and thermoneutrality similarly reduce the fat fraction within scBAT and sWAT. While we interpret that the cold exposure was sufficient to induce thermogenesis, we suggest that an increased lipolytic activity within adipocytes, as a consequence of fasting, may be the primary cause of the decreased fat fraction in both sWAT and scBAT in our study. The current study highlights the potential influence of fasting on the fat fraction in scBAT and stresses the importance of inclusion of a thermoneutral control group in studies investigating the BAT‐modulating effect of cold exposure.

## Graphical abstract

In young, lean, 12 h‐fasted volunteers we showed that cold exposure reduces the fat fraction within supraclavicular brown adipose tissue (scBAT) and subcutaneous white adipose tissue (sWAT) as assessed by MRI. Interestingly however, we also observed a decrease at thermoneutrality. We suggest that increased lipolytic activity within adipocytes, as a consequence of fasting, may be the primary cause of this effect, stressing the importance of inclusion of a thermoneutral control group in studies investigating effects of cold exposure on fat fraction.

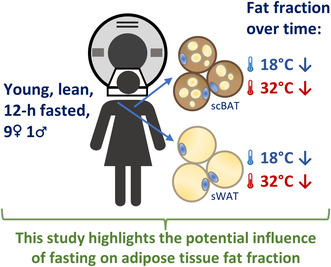

**Abbreviations**
BAT: brown adipose tissue
BMI: body mass index
FDG: fluorodeoxyglucose
FF: fat fraction
FTHA: fluoro‐6‐thia‐heptadecanoic acid
MRI: magnetic resonance imaging
PET‐CT: positron emission tomography‐computed tomography
scBAT: supraclavicular brown adipose tissue
SNS: sympathetic nervous system
sWAT: subcutaneous white adipose tissue
TRL: triglyceride‐rich lipoprotein
UCP‐1: uncoupling protein 1
WAT: white adipose tissue

## 1 INTRODUCTION

Brown adipose tissue (BAT) is a metabolically highly active tissue that dissipates energy stored within intracellular triglycerides as heat (i.e., thermogenesis). In humans, BAT is primarily located in the supraclavicular area and surrounding the large arteries. This is in contrast to white adipose tissue (WAT), which mainly stores energy within triglycerides and is located throughout the body. The main physiological activator of BAT thermogenesis is cold, which results in increased sympathetic nervous system (SNS) outflow towards, and β‐adrenergic activation of the tissue. This leads to increased intracellular lipolysis and subsequent combustion of fatty acids, the produced energy of which is dissipated as heat via uncoupling protein 1 (UCP‐1). In parallel, SNS activation also stimulates lipolytic activity within WAT, thereby fueling the increased energy demand in BAT in addition to providing fuel for other tissues. Interestingly, upon prolonged periods of SNS activation, WAT may acquire brown‐like features in a process called browning, giving rise to ‘beige’ adipocytes that are scattered between white adipocytes.^1^, ^2^

Nutrient uptake of BAT upon thermogenic activation is often used as an indirect measure for the tissue's activity. In fact, quantifying BAT activity by [^18^F]fluorodeoxyglucose (FDG) positron emission tomography‐computed tomography (PET‐CT) scans was used for the original discovery that metabolically active BAT is present in adult humans^3^ and is currently the gold standard for its visualization. However, a limitation of this method is that glucose uptake via glucose transporter 4 highly relies on insulin sensitivity, therefore less uptake of the [^18^F]FDG label occurs in case of insulin resistance. Furthermore, fatty acids rather than glucose are the primary energy source for thermogenesis.^4^ As an alternative, attempts have therefore been made to visualize BAT by measuring free fatty acid uptake using [^18^F]fluoro‐6‐thia‐heptadecanoic acid (FTHA) PET‐CT scans.^5^ Although the uptake of this tracer is not affected by insulin resistance, in the bloodstream fatty acids including FTHA efficiently bind to albumin for transport to the liver thereby hampering and thus underestimating its uptake by BAT and resulting in a strong hepatic background signal.^6^ More importantly, we previously reported that BAT preferentially takes up fatty acids from triglyceride‐rich lipoprotein (TRL)‐derived triglycerides rather than in free form bound to albumin in mice.^7^

As opposed to quantifying nutrient uptake, one may instead quantify substrate combustion for example using a [^15^O]O_2_ tracer with PET‐CT.^8^ However, a shared limitation between all these methods is the accompanying radiation‐burden, which restricts its use for repeated measurements in humans. BAT may instead be visualized by non‐invasive magnetic resonance imaging (MRI) which does not involve radioactivity and can therefore be safely used in humans, also for repeated measures. Specifically, distinctions can be made between MR signal derived from protons in fat versus in water using chemical shift based water‐fat separation, allowing for quantifying fat fraction changes within supraclavicular BAT (scBAT) as a measure of substrate combustion after lipolysis, and thus its activity.^9^

Using this method, others have previously reported reductions in fat fraction up to approx. −3% within scBAT upon cold exposure (e.g. [10, 11, 12, 13, 14, 15, 16]). However, these measurements were mostly done on two fixed time points (i.e., pre‐cooling and post‐cooling) or without using techniques that help minimizing motion artifacts. Furthermore, comparisons to a control group that was not exposed to cold are largely lacking. We therefore recently optimized a dynamic MRI protocol including breath‐holds, co‐registration and mutual thresholding to minimize motion‐induced variation, with which we were able to quantify dynamic changes in scBAT fat fraction in humans with a one‐minute time resolution.^17^ Using this technique, here, we aimed to study the effect of cold exposure versus thermoneutrality on changes in fat fraction of human scBAT.

## 2 MATERIALS AND METHODS

### 2.1 Participants

Ten volunteers of Europid descend aged 18–35 years and with a body mass index (BMI) of 18–25 kg/m^2^ were recruited via e‐mails and flyers. Exclusion criteria were medical conditions or the use of any medication known to affect lipid and/or glucose metabolism, smoking, recent excessive weight change and contra‐indications for MRI. Eligibility for inclusion was determined via a screening consisting of anthropometry, a medical history questionnaire, a questionnaire on MRI contra‐indications, and a 10 h fasted blood sample in which we determined amongst others serum glucose, insulin, total cholesterol and triglycerides via the routine hospital laboratory.

### 2.2 Study design

The study was approved by the Medical Ethical Committee of the Leiden University Medical Center (LUMC) and undertaken in accordance with the principles of the revised Declaration of Helsinki. Written informed consent was obtained from all participants prior to participation.

**FIGURE 1:**
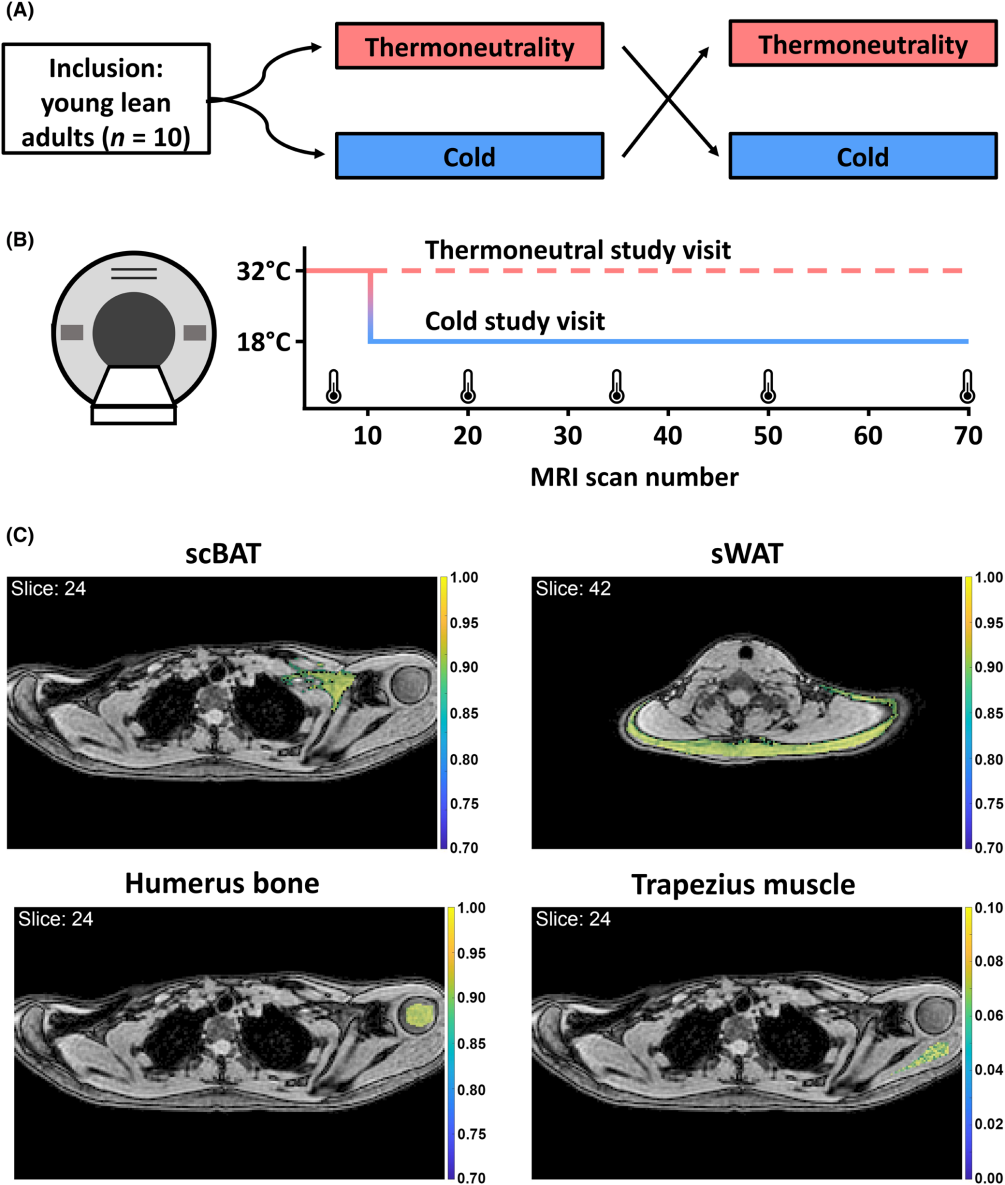
Study design. (A) Ten young lean adults were included in a cross‐over design study. (B) Participants were subjected to 70 consecutive magnetic resonance imaging (MRI) scans. For both study visits, blanket temperature was initially set at 32°C, and was either lowered to 18°C after 10 scans (i.e., cold exposure) or kept at this temperature (i.e., thermoneutrality). Self‐reported thermal perception was determined as indicated by the thermometers. (C) Regions of interest were delineated, within which voxels with pre‐defined fat fraction were selected. scBAT, supraclavicular brown adipose tissue; sWAT, subcutaneous white adipose tissue.

After inclusion as determined via the screening, participants visited the research center twice within a three‐week period in a cross‐over design (i.e., for a ‘cold’ and a ‘thermoneutral’ study visit, in no particular order; Figure 1A). Participants were instructed to fast 12 h before each study visit, to refrain from vigorous exercise 48 h prior to each study visit and to avoid drinking alcohol or caffeinated drinks 24 h prior to each study visit. In addition, participants were asked to wear a t‐shirt and shorts during the study visits. Study visits took place between April 2022 and September 2023. At the start of each study visit, a catheter was inserted in the antecubital vein for repeated venous blood sampling. Next, the participants were either positioned in an MRI scanner under one (*n* = 5) or in between (*n* = 5) two temperature controlled water circulating blanket(s) (Blanketrol III hyper‐hypothermia system; Cincinnati Sub‐Zero, Cincinnati, OH, USA). After 10 min of postural adaptation, up to 70 MRI scans were acquired as detailed below between approx. 10 a.m. and 12 p.m. clock time. Water temperature of the water circulating blankets was set at 32°C and was lowered to and maintained at 18°C after 10 scans, corresponding with a cold exposure, or was kept constant at 32°C for the entire duration of the scans (Figure 1B). Blood was drawn prior to the first scan, and after scan 5, 20 and 70 (i.e., on average after 9, 35 and 100 min); however, these samples appeared not suitable for analysis as described below. Thermal perception scores were self‐reported after scan 5, 20, 35, 50 and 70 using a numeric rating scale, with 1 corresponding with feeling “comfortable” and 10 with feeling “extremely cold”. The experiment would be stopped in case of self‐reported shivering, but this did not occur in any of the participants.

The repeated blood withdrawals appeared technically very challenging because participants were positioned horizontally in the scanner and could not move freely, and the cold exposure caused peripheral vasoconstriction. As a consequence, the average time required for each blood withdrawal was 6.6 ± 3.4 min, and at each time point blood could not be drawn in ≥4 study visits, rendering measurements of glucose, insulin, total cholesterol, triglycerides and free fatty acids within the blood samples unsuitable for analysis. Times and durations of blood withdrawals are shown in Table S1.

### 2.3 MRI image acquisition and analysis

MRI data were acquired as described previously.^17^ Briefly, 70 scans were acquired at 3T (Philips Ingenia Elition X, *n* = 3 participants or Philips Ingenia X, *n* = 7 participants; Philips Healthcare, Best, the Netherlands) using a 16‐channel head‐and‐neck coil, a 12‐channel phased array placed on top of the subject, and an in‐table 16‐channel array for signal reception. A 3D gradient‐echo 12‐point chemical shift based water‐fat separation (mDIXON Quant) sequence with the following parameters was used: repetition time (TR) = 12 ms, first echo time (TE) = 1.12 ms, echo time separation ΔTE = 0.87 ms, flip angle = 3°, field of view (FOV) = 400 × 229 × 134 mm^3^ (right–left, feet–head, anterior–posterior), isotropic resolution = 2.1 mm, and breath‐hold time = 16 s. Philips SENSE (P reduction (AP) = 2.3; S reduction (FH) = 1.3) was used as acceleration technique. The average acquisition time per scan was 1.15 and 1.38 min for scans performed on the Philips Ingenia Elition X and Philips Ingenia X, respectively, requiring 2 breath‐holds of 17 s each per acquisition. Voxel‐based water and fat signals were separated using an in‐house‐developed complex‐based fitting algorithm.^17^

To obtain fat fraction changes within the scBAT, trapezius muscle, humerus bone and subcutaneous WAT (sWAT) over time, regions of interest were manually coarsely delineated in the first scan (i.e., reference scan). First‐echo magnitude images of each subsequent scan were next co‐registered to this reference scan using Elastix,^18^ allowing the same regions of interest to be used for all scans. We next applied a mutual thresholding approach by only including voxels with a pre‐defined fat fraction (i.e., >30% for scBAT, the humerus bone and subcutaneous white adipose tissue and ≤10% for the trapezius muscle; Figure 1C) in both the scan of interest and the reference scan, and determined the voxel‐wise fat fraction differences between the scan of interest and the reference scan as described before.^17^

### 2.4 Statistical methods

Pearson product–moment correlation coefficients were used to determine correlations between the change in fat fraction (in scans 11 through 70) with time. Fat fraction changes from baseline (i.e., from the first scan) were determined by two‐sided one‐sample t‐test after averaging the changes of the last five scans where *n* ≥ 8 in both temperature conditions. Fat fraction changes were also determined after averaging the changes of the last ten scans, and comparing that to the average of the first ten scans, however this did not change the interpretation of the data and these analyses are therefore not shown. Comparisons between groups (i.e., correlation coefficients and changes from baseline) and between tissues (i.e., changes from baseline) were made by two‐sided paired and independent sample *t*‐tests, respectively. Differences in thermal perception scores were determined by Wilcoxon signed‐rank test. *p* values less than .05 were considered statistically significant. Data are presented as mean ± standard error of the mean (SEM).

## 3 RESULTS

Ten young and lean adults (mean age: 21.5 ± 0.7 years; mean BMI: 21.7 ± 0.5 kg/m^2^), of which nine females and one male, participated in this study. Participants were normoglycemic and normoinsulinemic (mean fasting glucose: 4.8 ± 0.1 mM; mean fasting insulin 10.6 ± 1.6 mIU/L), and showed no signs of dyslipidemia (mean total cholesterol: 4.2 ± 0.2 mM; mean triglycerides: 0.8 ± 0.1 mM). In 7 out of 20 study visits, the experiment was stopped on average after 57 scans (i.e., on average 89 min), because time limits were reached (*n* = 6) or the participant required urination (*n* = 1).

**FIGURE 2:**
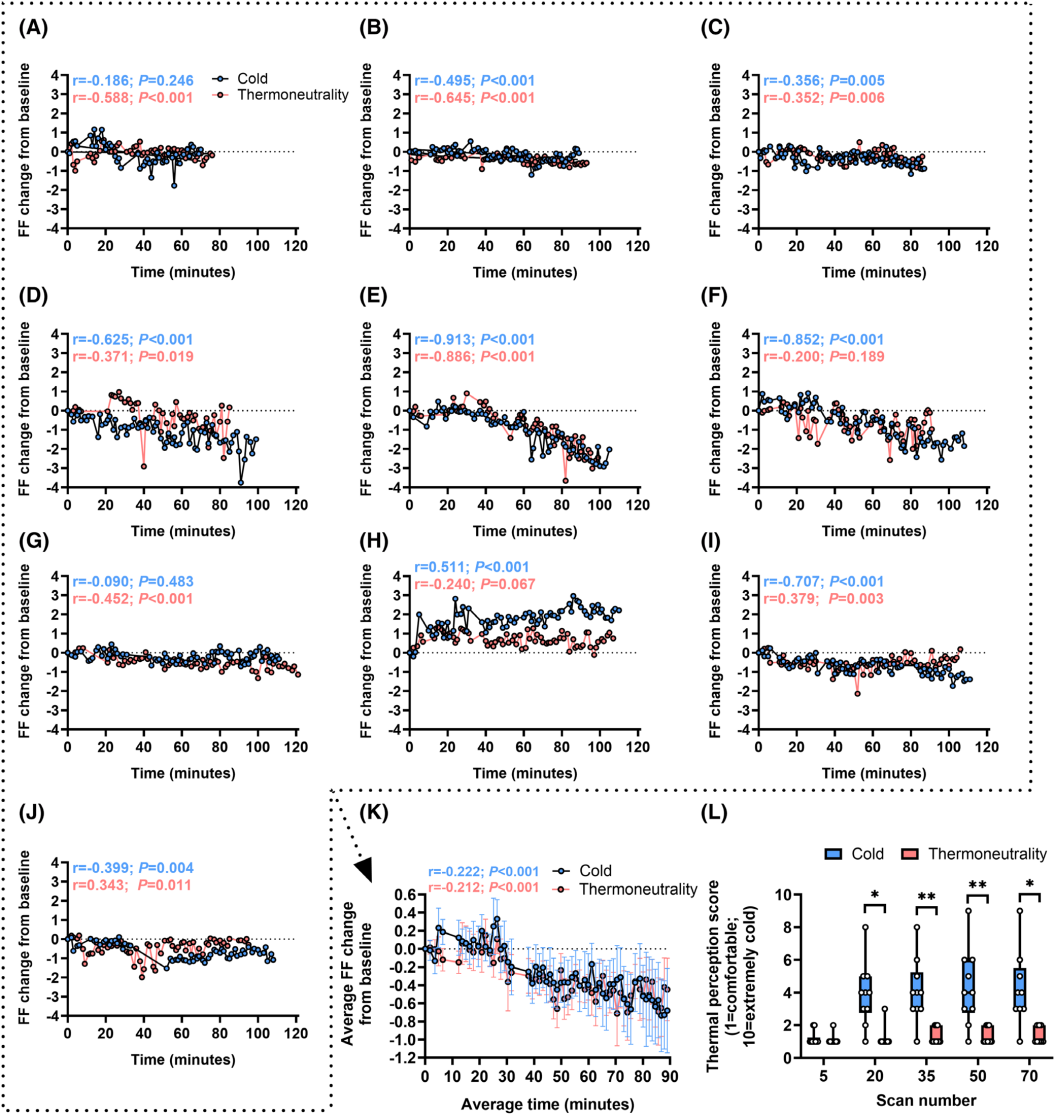
Cold exposure and thermoneutrality similarly reduce the fat faction of supraclavicular brown adipose tissue. In ten young and lean adults, supraclavicular brown adipose tissue fat fraction (FF) change from baseline was determined upon cold exposure and upon thermoneutrality in up to 70 consecutive magnetic resonance imaging (MRI) scans. To this end, participants were positioned (A–E) under one, or (F–J) in between two temperature controlled water circulating blanket(s), and data of each individual participants was plotted. (K) Data of participants in A–J was averaged for scans where *n* ≥ 8. (L) Thermal perception scores were self‐reported using a numeric rating scale. (A, B, D–J) female. (C) male. (K) data are presented as mean ± SEM. (L) boxes extend from the 25th to 75th percentile; line indicates the median; whiskers are plotted down to the minimum and up to the maximum value. *p < .05, **p < .01.

Similar to our previous observations,^17^ the fat fraction in scBAT decreased over time upon cold exposure (i.e., from scan 11 onwards). Specifically, the fat fraction in 7 out of 10 participants showed a significant negative correlation with time (Figure 2A–J). Interestingly however, we could also observe decreased fat fractions over time upon thermoneutrality (Figure 2A–J), and after combining data of all participants, we were unable to observe differences (*p* = .55) in the negative correlations between the two temperature conditions (cold: *r* = −.222, *p* < .001; thermoneutrality: *r* = −.212, *p* < .001; Figure 2K), nor (*p* = .78) in the overall reductions in fat fraction from baseline (cold: −0.67 ± 0.41%, *p* < .001; thermoneutrality: −0.60 ± 0.28%, *p* < .001). This effect was not explained by a lack of cold exposure, as self‐reported thermal perception was consistently higher (i.e., colder) upon cold exposure after scan 20, 35, 50 and 70 (median 4/10 versus 1/10), while scores were identical at baseline (i.e., after scan 5) (Figure 2L).

**FIGURE 3:**
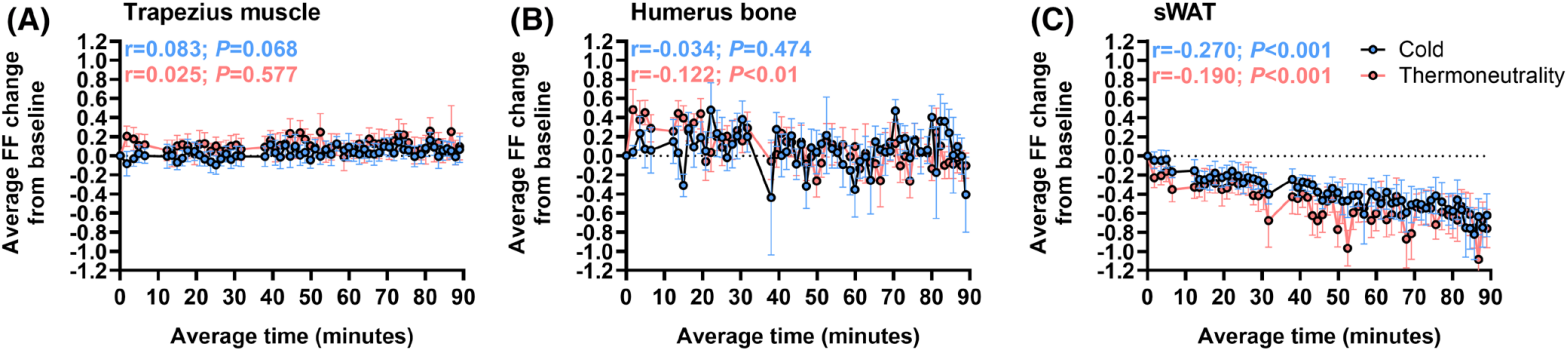
Cold exposure and thermoneutrality do not affect fat fraction in the trapezius muscle and humerus bone, while reducing fat fraction in subcutaneous white adipose tissue. In ten young and lean adults, fat fraction (FF) change from baseline was determined in the (A) trapezius muscle, (B) the humerus bone, and in (C) subcutaneous white adipose tissue (sWAT) upon cold exposure and upon thermoneutrality in up to 70 consecutive magnetic resonance imaging (MRI) scans. Data of the individual participants was averaged for scans where *n* ≥ 8. Data are presented as mean ± SEM.

We next quantified the fat fraction changes in the trapezius muscle and the humerus bone as control tissues. Indeed in the trapezius muscle, we could not observe a change in the fat fraction over time in either temperature condition (cold: *r* = .083, *p* = .068; thermoneutrality: *r* = .025, *p* = .577; Figure 3A). As expected, in the humerus bone the variation in the fat fraction appeared larger than in the trapezius muscle due to the heterogenic structure of the bone tissue. While we could observe a negative correlation in the humerus bone only upon thermoneutrality (*r* = −.122, *p* < .01), the fat fraction change in the last scans did not differ from baseline in both temperature conditions (cold: −0.01 ± 0.21%, *p* = .43; thermoneutrality: −0.03 ± 0.13%; *p* = .26; Figure 3B). In contrast, the fat fraction in sWAT decreased over time both upon cold exposure (*r* = −.270, *p* < .001) and upon thermoneutrality (*r* = −.190, *p* < .001), with no observed difference (*p* = .92) between the two temperature conditions (Figure 3C). Similar as in scBAT, in sWAT we also found no differences (*p* = .76) in the overall reductions in fat fraction from baseline between the two temperature conditions (cold: −0.72 ± 0.21%, *p* < .001; thermoneutrality: −0.73 ± 0.19%, *p* < .001). The fat fraction reductions upon cold exposure and upon thermoneutrality also did not differ from those in scBAT (*p* = .85 and *p* = .74, respectively).

## 4 DISCUSSION

The effect of cold on BAT fat fraction has often been studied, showing decreases compared with baseline (i.e., pre‐cooling). However, comparisons to a control group that was not exposed to cold are largely lacking. Therefore in the current study, we investigated the effects of cold exposure on fat fraction changes in scBAT compared to a thermoneutral control condition. To our surprise, we found that the fat fraction within scBAT decreases over time both upon cold exposure and during thermoneutrality, with no observed differences between both conditions. Moreover, we could observe similar reductions over time in the fat fraction of sWAT, but not of the trapezius muscle nor the humerus bone.

Our finding that fat fraction within scBAT decreased over time upon cold exposure is in line with previous reports (e.g. [10, 11, 12, 13, 14, 15, 16]). Overall, our effect size seems smaller than those observed in most previous studies. This may be explained by differences in the protocol used as we detailed previously^17^; an additional factor may be that we mainly included females in our study. A striking finding of the current study is that we found a similar reduction in scBAT fat fraction over time in a thermoneutral control condition, which is in line with effects that Gashi et al.^15^ observed in some of their participants. Given that self‐reported thermal perception was consistently higher upon cold exposure, we interpret that the similarity between both temperature conditions in our study was not due to a lack of cold exposure. Instead, a common factor between both temperature conditions is that MRI's were performed on fasted subjects. Fasting is known to stimulate intracellular lipolysis in white adipocytes e.g., via hormonal changes and an increase in sympathetic nervous system activity.^19^ Indeed, we observed a decrease in fat fraction in sWAT in both temperature conditions, suggesting ongoing lipolytic activity within WAT, and this effect was strikingly similar to that within scBAT. While some rodents such as mice have very distinct patches of BAT, human BAT morphologically more resembles that of beige adipose tissue, consisting of brown adipocytes interspersed between white adipocytes (e.g. [3]). Importantly, the image resolution obtained by chemical shift based water‐fat separation MRI scans do not allow for distinguishing brown and white adipocytes within scBAT. We therefore suggest that an increased lipolytic activity within white adipocytes, as a consequence of fasting, may be the cause of the decreased fat fraction in both sWAT and scBAT in our study. Fasting may also at least in part explain effects observed previously by others as fasting periods ranging between 4 h and >10 h have been reported (e.g. [10, 12, 13, 15, 16, 20, 21, 22, 23]), whereas other studies left the fasting status unreported (e.g. [11, 14]). This fasting effect may thus be obscuring any effect on fat fraction that thermogenic activity within brown adipocytes following cold exposure may have had. While an intuitive next step would be an attempt to mitigate the potential effect of fasting e.g., by giving subjects a standardized breakfast prior to each study visit, this may in fact stimulate (BAT‐associated) diet‐induced thermogenesis, causing the effects of cooling to be underestimated.^24^, ^25^

Previously,^17^ we showed that our MRI protocol allows for quantifying the fat fraction within scBAT with a low intrasubject variability, given that it incorporates breath‐holds and techniques such as co‐registration and mutual thresholding that help minimizing potential motion artifacts. The absence of fat fraction changes within control tissues (i.e., the trapezius muscle and the humerus bone) indeed confirms that the observed changes in fat fraction in scBAT and sWAT are not caused by a potential technical drift in our data.

In conclusion, we show that in fasted, young and lean individuals the fat fraction within scBAT and sWAT decreases over time both upon cold exposure and during thermoneutrality. The current study highlights the potential influence of fasting on the fat fraction in scBAT and stresses the importance of inclusion of a thermoneutral control group.

## AUTHOR CONTRIBUTIONS

Conceptualization: Robin van Eenige, Aashley S. D. Sardjoe Mishre, Borja Martinez‐Tellez, Patrick C. N. Rensen, Hermien E. Kan. Data Curation: Robin van Eenige, Aashley S. D. Sardjoe Mishre. Formal Analysis: Robin van Eenige. Funding Acquisition: Mariëtte R. Boon, Patrick C. N. Rensen, Hermien E. Kan. Investigation: Robin van Eenige, Carlijn A. Hoekx, Aashley S. D. Sardjoe Mishre, Maaike E. Straat. Methodology: Aashley S. D. Sardjoe Mishre, Borja Martinez‐Tellez, Hermien E. Kan. Project Administration: Mariëtte R. Boon, Hermien E. Kan. Software: Robin van Eenige, Aashley S. D. Sardjoe Mishre. Supervision: Mariëtte R. Boon, Borja Martinez‐Tellez, Patrick C. N. Rensen, Hermien E. Kan. Validation: Robin van Eenige, Aashley S. D. Sardjoe Mishre. Visualization: Robin van Eenige. Writing—Original Draft Preparation: Robin van Eenige. Writing—Review & Editing: Robin van Eenige, Aashley S. D. Sardjoe Mishre, Carlijn A. Hoekx, Mariëtte R. Boon, Borja Martinez‐Tellez, Patrick C. N. Rensen, Hermien E. Kan.

## FUNDING INFORMATION

This work was supported by the Leiden University Medical Center (LUMC profile area “biomedical imaging” grant to H.E.K. and P.C.N.R), by an NWO‐Veni (09150161910073 to M.R.B.), by the Netherlands Cardiovascular Research Initiative: an initiative with support of the Dutch Heart Foundation (CVON2017 GENIUS‐2 to P.C.N.R) and by the Dutch and British Heart Foundation (02–001‐2021‐B020 to M.R.B.).

## DISCLOSURES

H.E.K. reports research support from Philips Healthcare during the conduct of the study; no personal financial benefits were received. The remaining authors declare no conflict of interest.

## Supporting information


Table S1.

## Data Availability

The datasets used and/or analyzed during the current study are available from the corresponding author on reasonable request.
