# Supplementary material for: Cold exposure and thermoneutrality similarly reduce supraclavicular brown adipose tissue fat fraction in fasted young lean adults
Source: FASEB J. 2025 Jan 11;39(1):e70307. doi: 10.1096/fj.202402415R (PMC11724390; doi:10.1096/fj.202402415R)
Supplement: Supplementary file 1 — Table S1. [file FSB2-39-e70307-s001.pdf]

Supplemental data

Cold exposure and thermoneutrality similarly reduce supraclavicular brown adipose tissue fat fraction in fasted young lean adults

Robin van Eenige[a,b,c], Carlijn A. Hoekx[a,b], Aashley S.D. Sardjoe Mishre[c], Maaike E. Straat[a,b], Mariëtte R. Boon[a,b], Borja Martinez-Tellez[a,b,d,e,f], Patrick C.N. Rensen[a,b], Hermien E. Kan[c]

[a]Division of Endocrinology, Department of Medicine, Leiden University Medical Center, Leiden, The Netherlands

[b]Einthoven Laboratory for Experimental Vascular Medicine, Leiden University Medical Center, Leiden, The Netherlands

[c]Department of Radiology, C.J. Gorter MRI Center, Leiden University Medical Center, Leiden, The Netherlands.

[d]Department of Nursing Physiotherapy and Medicine, SPORT Research Group (CTS-1024), CIBIS Research Center, University of Almería, Almería, Spain

[e]Biomedical Research Unit, Torrecárdenas University Hospital, 04009 Almería, Spain

[f]CIBER de Fisiopatología de la Obesidad y Nutrición (CIBEROBN), Instituto de Salud Carlos III, Granada, Spain

Supplementary Table 1. Times and durations of blood withdrawals. Ten young lean adults were included in a cross-over design study and were subjected to 70 consecutive magnetic resonance imaging (MRI) scans upon two temperature conditions. Blood was drawn just prior to the first scan, and after scan 5, 20 and 70. The time (from start) and duration of blood withdrawals were estimated from timestamps in MRI metadata. N/A, not applicable.

Participant	***Temperature condition***	***Scan 5***		***Scan 20***		***Scan 70***
		**Time (hh:mm:ss)**	**Duration (mm:ss)**	**Time (hh:mm:ss)**	**Duration (mm:ss)**	**Time (hh:mm:ss)**
1	**Cold**	00:08:19	06:59	00:32:31	09:16	N/A
	**Thermoneutrality**	00:07:54	06:10	00:29:15	04:15	01:16:43
2	**Cold**	00:07:59	06:19	00:29:47	04:55	01:29:03
	**Thermoneutrality**	00:08:54	08:10	00:32:58	07:46	01:33:14
3	**Cold**	00:07:43	03:42	00:28:18	04:57	01:26:57
	**Thermoneutrality**	00:07:16	04:16	00:28:47	03:25	01:25:54
4	**Cold**	N/A	N/A	N/A	N/A	N/A
	**Thermoneutrality**	N/A	N/A	N/A	N/A	N/A
5	**Cold**	N/A	N/A	N/A	N/A	N/A
	**Thermoneutrality**	00:12:45	13:11	00:49:25	07:57	N/A
6	**Cold**	00:08:28	04:54	00:33:57	04:24	01:48:05
	**Thermoneutrality**	00:09:00	05:56	00:33:57	05:19	01:30:58
7	**Cold**	00:08:45	03:45	00:36:40	12:17	01:51:04
	**Thermoneutrality**	00:14:06	13:06	00:45:53	06:54	02:09:21
8	**Cold**	00:10:18	01:20	00:34:42	06:43	01:49:44
	**Thermoneutrality**	00:09:45	05:53	00:35:19	06:12	01:46:39
9	**Cold**	00:08:26	04:36	00:33:27	04:31	01:50:37
	**Thermoneutrality**	00:08:16	04:28	00:33:52	07:37	01:45:50
10	**Cold**	00:09:19	06:18	00:42:32	18:26	01:48:12
	**Thermoneutrality**	N/A	N/A	N/A	N/A	01:35:20
